# A cross-sectional study on the correlation between cytokines in a pelvic environment and tubal factor infertility

**DOI:** 10.1186/s12884-020-03322-y

**Published:** 2020-10-22

**Authors:** Jiacong Yan, Chengbo Liu, Han Zhao, Chunyan Wang, Huimei Yao, Qiong Lu, Jia Liu, Yun Feng

**Affiliations:** 1 Department of Obstetrics and Gynecology, The First People’s Hospital of Yunnan Province, 650032 Kunming, China; 2 The Affiliated Hospital of Kunming University of Science and Technology, 650500 Kunming, China; 3 Jiang Ling County People’s Hospital, 434100 Jingzhou, China; 4 The second people’s Hospital of Baoshan City, 678000 Baoshan, China; 5 Cangyuan Wa Autonomous County People’s Hospital, 677400 Cangyuan, China

**Keywords:** Pelvic environment, Cytokine, Infertility, Etiology, Tubal factor infertility

## Abstract

**Background:**

This cross-sectional study aimed to evaluate the levels of tumor necrosis factor-alpha (TNF-ɑ), interleukin-8 (IL-8), interleukin-6 (IL-6), and transforming growth factor-beta 1 (TGF-β1) in patients with primary and secondary tubal factor infertility (TFI) compared with fertile subjects, and to compare immune indexes in the serum and peritoneal fluid samples obtained from patients with TFI.

**Methods:**

The pelvic fluid and peripheral blood of patients with TFI diagnosed by hysteroscopy and laparoscopy were taken as the study objects. The pelvic fluid and peripheral blood of patients who underwent hysteromyomectomy at the same time were taken as the control group. The contents of TNF-ɑ, IL-8, IL-6, and TGF-β1 in serum and peritoneal fluid were determined by enzyme-linked immunosorbent assay, and the levels of these cytokines in serum and pelvic fluid were compared between the two groups.

**Results:**

Patients with secondary TFI showed significantly higher levels of TNF-ɑ, IL-8, IL-6 and TGF-β1 in the serum (26.15 ± 3.51 vs. 19.61 ± 0.157, 32.18 ± 15.13 vs. 5.73 ± 1.99, 38.84 ± 3.46 vs. 30.48 ± 0.61, and 38.37 ± 3.14 vs. 32.25 ± 1.69, respectively) and peritoneal fluid samples (129.73 ± 183.4 vs. 34.63 ± 0.56, 111.44 ± 207.42 vs. 15.34 ± 0.41, 80.01 ± 109.91 vs. 15.67 ± 0.52, and 82.54 ± 115.99 vs. 45.34 ± 0.41, respectively) compared with the control group. Patients with primary TFI exhibited significantly elevated concentration of TNF-α, IL-8, IL-6 and TGF-β1 in the peritoneal fluid samples (36.88 ± 2.67 vs. 34.63 ± 0.56, 19.47 ± 3.51 vs. 15.34 ± 0.41, 80.01 ± 109.91 vs. 15.67 ± 0.52, and 82.54 ± 115.99 vs. 45.34 ± 0.41, respectively) when compared to the controls. In patients with secondary infertility, the levels of TNF-α (26.15 ± 3.51 vs. 129.73 ± 183.4), IL-8 (32.18 ± 15.13 vs. 111.44 ± 207.42), IL-6 (38.84 ± 3.46 vs. 80.01 ± 109.91) and TGF-β1 (38.37 ± 3.14 vs. 82.54 ± 115.99) in the serum were significantly lower than those in the peritoneal fluid, whereas no significant difference was observed in the primary TFI group between the serum and peritoneal fluid cytokines levels.

**Conclusion:**

The expression of cytokines in the pelvic environment of patients with TFI is upregulated compared to patients who do not have infertility issues. The detection of cytokines TNF-ɑ, IL-6, IL-8, and TGF-β1 in the pelvic fluid of tubal infertility patients can allow for further understanding of the etiology of TFI.

## Background

Infertility refers to couples who are unable to conceive a child despite having normal sexual lives without any contraceptive measures for one year [1]. Primary infertility means that a couple has never been able to conceive a pregnancy, while secondary infertility describes the inability to become pregnant after giving birth to one or more children. Infertility affects 60–80 million people worldwide, accounting for approximately 10–15% of the world’s reproductive-age population [2]. The WHO forecasts that in the 21st century, infertility will probably become one of the three major diseases affecting human health, next to cancer and cardiovascular diseases [3]. Infertility patients induced by female factors accounted for 45% [4]. A study revealed that approximately 70% of female infertility patients are induced by pelvic factors, from which the cases of tubal factor infertility (TFI) account for 60% [5]. TFI refers to the obstruction of the oviduct and fimbria caused by genital tract infection, which affects the binding and transport of eggs and sperm, ultimately leading to female infertility. Therefore, tubal factors are the main cause of female infertility. Due to chronic pelvic inflammation, tubal adhesion, stiffness, or being pulled, twisted, or occluded by surrounding scar tissue [6–8], the oviduct loses its physiological function of transporting sperm, eggs and fertilized eggs, resulting in infertility.

A recent study has revealed that [9] the implantation rate of patients with hydrosalpinx after treatment with assisted reproductive technology was significantly lower when compared to patients with male factor infertility. This may be due to the presence of large amounts of white blood cells and inflammatory cytokines in hydrosalpinx fluid, such as interleukin-8 (IL-8), interleukin-12 (IL-12) and tumor necrosis factor-alpha (TNF-α). After these cells and cytokines enter the uterus, the uterine cavity environment is no longer conducive for embryo implantation. It has been reported that IL-6 levels are abnormally elevated in peritoneal fluid of patients with pelvic adhesions [4]. Omurtag et al. [10] found that IL-6 increased the incidence of adhesion and promoted the accumulation of inflammatory cells and fibers in a rat model of pelvic adhesions. IL-8 may act as an autocrine factor in the endometrium, affecting neovascularization, endometrial cell attachment, and cell growth [11]. Previous data showed significantly elevated levels of TNF-α in follicular fluids, which was associated with the poor-quality oocytes and embryos [12]. An analysis in infertile women showed that the expression level of transforming growth factor-beta 1 (TGF-β1) in blocked fallopian tubes was independently and inversely correlated with the postoperative pregnancy outcomes [13]. Thus, material changes in the pelvic environment and abnormal structure and function of the oviduct may cause female infertility.

In the present study, we aimed to compare the levels of inflammatory cytokines in the abdominal fluid and serum samples of patients with TFI and patients with no fertility issues. We hypothesized that the cytokine levels in patients with primary and secondary TFI would be higher than those in fertile subjects. Our results might contribute to a better understanding of the correlation between TFI and the immune status in the pelvic environment.

## Materials and Methods

### Study subjects

Patients diagnosed with infertility by hysteroscopy and laparoscopy in the Department of Reproductive Gynecology of the First People’s Hospital of Yunnan Province from January 2018 to June 2018 were recruited in this cross-sectional study. The main pathological change of TFI is non-specific salpingitis. These patients were divided into two groups, secondary infertility group (*n* = 30) and primary infertility group (*n* = 30), based on their medical history. The pelvic fluid samples were collected immediately after the laparoscope entered the abdominal cavity. The peripheral blood was also collected from these patients. The control group was composed of 29 patients who underwent hysteromyomectomy at the same time. Uterine fibroids were diagnosed according to the diagnostic criteria of uterine fibroids released by the International Federation of Gynecology and Obstetrics (8th edition) [14]. All these patients were examined for reproductive tract infection and vaginal discharge abnormality before the operation and showed no signs of infection. None of them were diagnosed with other gynecological and non-gynecological pelvic diseases. The pelvic fluid and peripheral blood from hysteromyoma patients were also collected. The body mass index (BMI) was calculated for all patients.

### Inclusion and exclusion criteria

Patients who were diagnosed with female factor infertility before the operation, who were < 35 years old, had normal ovarian functions, and with hysteroscopic and laparoscopic surgery indications were included. Patients with congenital malformation of the genital tract, ovulation failure, ovarian cysts, immunodeficiency or other pelvic or gynecological diseases, and patients with severe diseases in the liver, kidney, cardiovascular, or hematopoietic systems were excluded. No patients had a history of abortion.

### Measurement of inflammatory cytokines in serum

Five mL blood samples were collected in the morning by venipuncture and centrifuged at 3000 rpm for 5 min. Then the supernatants were obtained and stored at -20ºC before analysis. The levels of inflammatory cytokines, including TNF-α, IL-8, IL-6, and transforming growth factor-β1 (TGF-β1), in the serum were analyzed using commercially available enzyme-linked immunosorbent assay (ELISA) kits (Proteintech Group) following the manufacturer’s instructions. Serum samples (1 mL) were diluted and tested in triplicate per condition. The detection limit for TNF-α, IL-8, IL-6, and TGF-β1 was 1.0 pg/mL, 1.0 pg/mL, 3.8 pg/mL, and 3.3 pg/mL, respectively. The optical density (OD) values were determined using an ELISA analyzer (Bio-Rad, Hercules, USA) and the results were shown as concentrations of cytokines (pg/mL). This experiment was repeated three times.

### Measurement of inflammatory cytokines in peritoneal fluid

Peritoneal fluid samples from all subjects were collected during laparoscopy in a sterile manner. The fluid samples were immediately centrifuged at 400 g for 10 minutes. The supernatants were transferred to -80ºC prior to analysis. The concentrations of TNF-α, IL-8, IL-6, and TGF-β1 in peritoneal fluid samples were detected using ELISA kits (Kelvin Testing Co., Ltd, Suzhou, China) according to the manufacturer’s instructions. Fluid samples (1 mL) were diluted and tested in triplicate per condition. The detection limit for TNF-α, IL-8, IL-6, and TGF-β1 was 4.69 pg/mL. The OD values were determined using an ELISA analyzer (Bio-Rad, Hercules, USA) and the results were shown as concentrations of cytokines (pg/mL). The cytokine expression levels were determined using an ELISA analyzer. Results were shown as concentrations of cytokines (pg/mL) in the peritoneal fluid samples. This experiment was repeated three times.

### Statistical analysis

Data were processed and analyzed using the statistical software SPSS 19.0. The means and standard deviations of relevant measurement data were calculated and expressed as mean ± standard deviations (x ± SD). The normally distributed clinical data were compared using independent sample *t*-tests, while non-normally distributed clinical data between groups were compared using Chi-squared tests. A value of *p* < 0.05 was considered statistically significant, while *p* < 0.01 was considered highly statistically significant.

### Ethics statement

All experimental protocols were approved by the Ethics Committee in the hospital and performed following the World Medical Association Declaration of Helsinki (Ethical committee approval number: KHLL2019-KY010). Both patients and their spouses were aware of the purpose of the operation, agreed to participate in the present study, and provided signed informed consent for the operation.

## Results

The age of patients in the primary and secondary infertility groups ranged within 24–33 years, with an average age of 30.45 ± 2.78 years old. The course of disease of patients in the two groups ranged within 2–10 years. The age of patients in the three groups was not statistically significant. The BMI values in the uterine fibroids (control) group, primary infertility, and secondary infertility group were 20.64 ± 1.77, 20.97 ± 2.00, and 20.71 ± 0.86, respectively, and they were not significantly different.

Compared to the patients with uterine fibroids, patients with secondary TFI showed significantly higher levels of TNF-α, IL-8, IL-6, and TGF-β1 in the serum samples, whereas the serum cytokine concentrations were not statistically significant between patients with primary TFI and the control group (Table 1). Both primary and secondary TFI groups showed significantly elevated levels of TNF-α, IL-8, IL-6, and TGF-β1 in the peritoneal fluid as compared to patients with uterine fibroids (Table 2).

**Table 1:** Comparison of the serum levels of TNF-α, IL-8, IL-6 and TGF-β1

	Fibroids group (*n *= 29)	Primary infertility group (*n* = 30)		Secondary infertility group (*n* = 30)	
			*p*-value		*p*-value
IL-8	5.73 ± 1.99	9.82 ± 1.69	1.03376E-11	32.18 ± 15.13^a^	5.55701E-39
IL-6	30.48 ± 0.61	33.52 ± 1.33	4.27837E-16	38.84 ± 3.46^a^	4.38063E-19
TGF-β1	32.25 ± 1.69	33.72 ± 0.67	4.61825-5	38.37 ± 3.14^a^	1.31313E-13
TNF-α	19.61 ± 0.157	21.48 ± 1.99	4.68514E-6	26.15 ± 3.51^a^	1.46452E-16

^a^indicates statistical significance between Secondary infertility group vs. Fibroids group

**Table 2:** Comparison of the levels of TNF-α, IL-8, IL-6 and TGF-β1 in peritoneal fluid

	Fibroids group (*n* = 29)	Primary infertility group (*n* = 27)		Secondary infertility group (*n* = 30)	
			*p*-value		*p*-value
IL-8	15.34 ± 0.41	19.47 ± 3.51^a^	2.71769E-7	111.44 ± 207.42^b^	8.7245E-20
IL-6	15.67 ± 0.52	20.08 ± 3.51^a^	9.62185E-7	80.01 ± 109.91^b^	4.74483-17
TGF-β1	45.34 ± 0.41	49.47 ± 3.51^a^	3.44894E-10	82.54 ± 115.99^b^	4.85473E-9
TNF-α	34.63 ± 0.56	36.88 ± 2.67^a^	1.50071E-5	129.73 ± 183.4^b^	1.45237E-22

^a^indicates statistical significance between Primary infertility group vs. Fibroids group

^b^indicates statistical significance between Secondary infertility group vs. Fibroids group

When we compared the levels of TNF-α, IL-8, IL-6, and TGF-β1 in the serum and peritoneal fluid samples in patients with secondary infertility, we found that the concentrations of all cytokines in the serum were significantly lower than those in the peritoneal fluid (Table 3). In the primary TFI group, no significant difference was observed in the cytokine levels obtained from serum and peritoneal fluid. The levels of TNF-α, IL-8, and TGF-β1 in the serum were slightly lower than those in the peritoneal fluid, while the serum IL-6 level was higher than that in the peritoneal fluid (Table 4).

**Table 3:** The levels of TNF-α, IL-8, IL-6 and TGF-β1 between the serum and peritoneal fluid in patients with secondary infertility

	Serum samples (*n *= 30)	Peritoneal fluid samples (*n *= 30)	*P*-value
IL-8	32.18 ± 15.13	111.44 ± 207.42^a^	6.1086E-21
IL-6	38.84 ± 3.46	80.01 ± 109.91^a^	1.3E-13
TGF-β1	38.37 ± 3.14	82.54 ± 115.99^a^	3.36E-14
TNF-α	26.15 ± 3.51	129.73 ± 183.4^a^	2.99E-30

^a^indicates statistical significance between serum level vs. peritoneal fluid level

**Table 4:** The levels of TNF-α, IL-8, IL-6 and TGF-β1 between the serum and peritoneal fluid in patients with primary infertility

	Serum samples (*n* = 30)	Peritoneal fluid samples (*n* = 27)	*P*-value
IL-8	9.82 ± 1.69	19.47 ± 3.51	4.14E-18
IL-6	33.52 ± 1.33	20.08 ± 3.51	2.54E-25
TGF-β1	33.72 ± 0.67	49.47 ± 3.51	2.03E-29
TNF-α	21.48 ± 1.99	36.88 ± 2.67	2.18E-35

We next compared the serum levels of TNF-α, IL-8, IL-6, and TGF-β1 between the primary and secondary infertility groups and found no significant differences (Table 5). The cytokine levels in peritoneal fluid samples were also not statistically significant between the primary and secondary TFI groups (Table 6).

**Table 5:** The serum levels of TNF-α, IL-8, IL-6 and TGF-β1 between patients with primary and secondary infertility

	Primary infertility group (*n* = 30)	Secondary infertility group (*n* = 30)	*P*-value
IL-8	9.82 ± 1.69	32.18 ± 15.13	1.51E-36
IL-6	33.52 ± 1.33	38.84 ± 3.46	4.39E-11
TGF-β1	33.72 ± 0.67	38.37 ± 3.14	1.528-11
TNF-α	21.48 ± 1.99	26.15 ± 3.51	1.02E-9

**Table 6:** The levels of TNF-α, IL-8, IL-6 and TGF-β1 in peritoneal fluid between patients with primary and secondary infertility

	Primary infertility group (*n* = 27)	Secondary infertility group (*n* = 30)	*P*-value
IL-8	19.47 ± 3.51	111.44 ± 207.42	2E-22
IL-6	20.08 ± 3.51	80.01 ± 109.91	9.15E-19
TGF-β1	49.47 ± 3.51	82.54 ± 115.99	2.66E-9
TNF-α	36.88 ± 2.67	129.73 ± 183.4	5.26E-26

## Discussion

Female infertility may be caused by the abnormal changes of the immunoactive substances in the pelvic environment [15, 16]. The results of the present study revealed that the serum levels of TNF-α, IL-8, IL-6, and TGF-β1 were higher in the secondary group than in the uterine fibroids group, and the differences in the levels of TNF-α, IL-6, IL-8 and TGF-β1 in serum between these two groups were statistically significant. Cytokines such as IL-8, IL-10, and IL-6 can promote the local proliferation of fibroblasts, collagen deposition, and fibrinogenesis, ultimately causing the occurrence of pelvic cell fibrosis. This is the common mechanism of adhesion between tissues. Huihui Chou et al. reported that [17] patients with TFI generally have a longer duration of illness and that inflammatory response often occurs only in the genital tract. Therefore, this systemic response is not prominent, and the immune response to the body would not be significant. A study revealed that [18] in the early stage of abdominal surgery, the levels of TNF-α, IL-6, and interleukin-1 (IL-1) in the peritoneal fluid were relatively high, and these were much higher in the peritoneal fluid than in serum. This result suggests that these cytokines may mainly come from the peritoneal fluid of the patient. Consistently, our study revealed that when the levels of cytokines between the peritoneal fluid and serum of secondary TFI patients were compared, the levels of TNF-α, IL-8, IL-6, and TGF-β1 were higher in the peritoneal fluid than in serum.

We also showed that the levels of cytokines in peritoneal fluid from patients with TFI were higher than those in patients with uterine fibroids. The reason may be that the stimulatory effects of cytokines on the body mainly include participating in the process of proliferation and differentiation of immune cells, stimulating the growth of inflammatory cells, promoting antigen presentation, increasing the release of inflammatory mediators, and inducing the cascade reactions of other related cytokines. Further investigations will be needed to elucidate the mechanisms underlying the effect of these cytokines on TFI. As previously reported, the serum levels of TNF-α and IL-6 were higher in the secondary infertility group compared to the uterine fibroids group. The serum levels of IL-8 and TGF-β1 in the secondary infertility group were also higher than those in patients with uterine fibroids. These findings suggested that IL-8 and TGF-β1 also had diagnostic values for TFI.

Furthermore, we found that the levels of cytokines were higher in the secondary infertility group than in the primary infertility group. Wei Zhang et al. reported that [19] abortion could significantly increase the risks of ascending chlamydia infection and bacterial vaginosis. A study reported that [20] postpartum infection accounted for 12.45% of tubal obstructions. Ling Tang reported that [21] women who had a drug abortion were more prone to tubal obstruction than those who had an induced abortion. Also, abdominal surgery may cause an interruption of the continuity of the peritoneum, accompanied by bleeding, ischemia, foreign body stimulation (talcum powder on sutures and gloves, etc.), and tubal inflammation. This subsequently leads to tubal obstruction, umbrella atresia, or hydrosalpinx, and eventually leads to infertility. Rong Zhao et al. revealed that [22] the main causes of pelvic adhesions in infertility patients include a history of ectopic pregnancy, abortion, pelvic diseases, and uterine cavity operations. In the current study, as no patients had a history of abortion, the elevated cytokine levels in the secondary infertility group might be correlated to previous ectopic pregnancies, uterine cavity operations, or other pelvic diseases.

The present study has some limitations. First, the sample size was small and immune indicators selected for detection might not be the key cytokines involved in tubal factor infertility. Second, we did not take into account other factors that might be related to the inflammation levels of these patients such as leukocyte count, signs of endometriosis, history of smoking or diabetes mellitus, etc., which might potentially influence the concentration of cytokine. Third, future studies using other cytokine detection methods such as western blot and real-time PCR are needed to confirm the current results. To sum up, it is necessary to expand the number of cases, select more sensitive immune indices, and consider other inflammatory factors in further investigations.

## Conclusions

In conclusion, the present study revealed that the detection of local immune factors was more sensitive than that of systemic immune factors. However, since the collection of samples can cause trauma to patients, this cannot be easily achieved in clinics. Therefore, the detection of relevant immune indices, such as serum cytokines TNF-α, IL-6, IL-8, and TGF-β1, in patients with TFI would be more suitable as an auxiliary examination method to understand the condition of the TFI.

## Data Availability

The datasets used and/or analysed during the current study available from the corresponding author on reasonable request.

## Abbreviations

WHO: World Health Organization.
TFI: Tubal factor infertility.
TNF-α: Tumor necrosis factor-alpha.
TGF-β1: Transforming growth factor-β1.
ELISA: Enzyme-linked immunosorbent assay.

## References

[1] Gurunath S, Pandian Z, Anderson RA, Bhattacharya S. Defining infertility–a systematic review of prevalence studies. Hum Reprod Update. 2011;17(5):575–88. doi: 10.1093/humupd/dmr015. 10.1093/humupd/dmr015 21493634

[2] Tournaye HJ, Cohlen BJ (2012). Management of male-factor infertility[J]. Best practice research Clinical obstetrics gynaecology.

[3] Huang XJ, Zhang GQ, Qu HW, Kang GQ, Wang CJ, Suo L (2010). Infertility is about prevention[J]. China Practical Medicine.

[4] Lindsay TJ, Vitrikas KR (2015). Evaluation and treatment of infertility[J]. Am Family Phys.

[5] Wang XD.Clinical analysis of combined diagnosis and treatment of infertility in 117 women with laparoscope[J].Guide of China Medicine,2013(13):258–259.

[6] Lazer T, Meltzer S, Saar-Ryss B, Liberty G, Rabinson Y, Friedler S (2016). The place of selective hysterosalpingography and tubal canalization among sub-fertile patients diagnosed with proximal tubal occlusion[J]. Archives of gynecology obstetrics.

[7] Dreyer K, Lier MC, Emanuel MH, Twisk JW, Mol BW, Schats R (2016). Hysteroscopic proximal tubal occlusion versus laparoscopic salpingectomy as a treatment for hydrosalpinges prior to IVF or ICSI: an RCT[J]. Human reproduction.

[8] Calzolari S, Cozzolino M, Castellacci E, Dubini V, Farruggia A, Sisti G. Hysteroscopic Management of Uterine Arteriovenous Malformation[J]. JSLS: Journal of the Society of Laparoendoscopic Surgeons 2017, 21(2). 10.4293/JSLS.2016.00109 PMC5385144 28439193

[9] Yoshii N, Hamatani T, Inagaki N, Hosaka T, Inoue O, Yamada M (2013). Successful implantation after reducing matrix metalloproteinase activity in the uterine cavity [J]. Reprod Biol Endocrinol.

[10] Omurtag K, Grindler NM, Roehl KA, Bates GW, Beltsos AN, Odem RR (2012). How members of the Society for Reproductive Endocrinology and Infertility and Society of Reproductive Surgeons evaluate, define, and manage hydrosalpinges[J]. Fertility sterility.

[11] Arici A (2002). Local cytokines in endometrial tissue: the role of interleukin-8 in the pathogenesis of endometriosis. Ann N Y Acad Sci.

[12] Lee KS, Joo BS, Na YJ, Yoon MS, Choi OH, Kim WW (2000). Relationships between concentrations of tumor necrosis factor-alpha and nitric oxide in follicular fluid and oocyte quality. J Assist Reprod Genet.

[13] Li Z, Sun Y, Min W, Zhang D. Correlation between overexpression of transforming growth factor-beta 1 in occluded fallopian tubes and postsurgical pregnancy among infertile women. Int J Gynaecol Obstet. 2011 Jan;112(1):11–4. doi: 10.1016/j.ijgo.2010.07.016. Epub 2010 16. 10.1016/j.ijgo.2010.07.016 20837351

[14] Xie X,Gou WL.Obstetrics and Gynecology[M].Beijing: People’s medical publishing house,2013,8:310–313.

[15] Alijotas-Reig J, Esteve-Valverde E, Ferrer-Oliveras R, Llurba E, Gris JM (2017). Tumor Necrosis Factor-Alpha and Pregnancy: Focus on Biologics. An Updated and Comprehensive Review[J]. Clin Rev Allergy Immunol.

[16] Atzeni F, Grillo E, Masala IF, Sarzi-Puttini P, Jones GT (2016). Do Anti-TNF Blockers Increase the Risk of Lung Involvement in Patients with Ankylosing Spondylitis or Psoriatic Arthritis? A Systematic Review[J]. The Israel Medical Association journal: IMAJ.

[17] Liao HH. Relationship Between TCM Syndrome Pattern and Immuno-endocrine Function in Patients with Infertility Induced by Chronic Pelvic Inflammation[J]. J Guangzhou University Traditional Chin Med. 2006(1):29–31.

[18] Veaute C, Furlong LI, Cameo M, Harris JD, Vazquez-Levin MH (2009). Antiacrosin antibodies and infertility.II. Gene immunization with human proacrosin to assess the effect of immunity toward proacrosin/acrosin upon protein activities and animal fertility[J]. Fertility sterility.

[19] Zhang W, Xia HX.Etiology and epidemiology of tubal infertility[J]. Journal of Practical Obstetrics and Gynecology 2011(8):561–563.

[20] Qian RY, Wu X, Sheng J, Zheng P, Zhou Q, Duan AH (2017). [Evaluation of endometriosis fertility index in follow-up treatment of endometriosis combined with infertility patients after laparoscopic surgery][J]. Zhonghua fu chan ke za zhi.

[21] Tang L. Clinical analysis of 369 cases of secondary infertility after abortion[J]. MEDICAL JOURNAL OF WEST CHINA 2009(1):64–64,66.

[22] Zhao R, Ha CF (2016). Investigation of the influence factors of pelvic adhesion and its effect on fallopian tube retransmission in infertility patients[J]. Maternal & Child Health Care of China.

