# Correction to: A cross-sectional study on the correlation between cytokines in a pelvic environment and tubal factor infertility

**DOI:** 10.1186/s12884-021-04273-8

**Published:** 2021-12-23

**Authors:** Jiacong Yan, Chengbo Liu, Han Zhao, Chunyan Wang, Huimei Yao, Qiong Lu, Jia Liu, Yun Feng

**Affiliations:** 1 Department of Obstetrics and Gynecology, The First People’s Hospital of Yunnan Province, Kunming, 650032 China; 2 The Affiliated Hospital of Kunming University of Science and Technology, Kunming, 650500 China; 3 Jiang Ling County People’s Hospital, Jingzhou, 434100 China; 4 The second People’s Hospital of Baoshan City, Baoshan, 678000 China; 5 Cangyuan Wa Autonomous County People’s Hospital, Cangyuan, 677400 China

**Correction to: BMC Pregnancy Childbirth 20, 644 (2020)**

**https://doi.org/10.1186/s12884-020-03322-y**

Following publication of the original article [1], the authors reported an error in the Results and additional supporting funding in the Funding section.

In the Results section, paragraph must read:

Compared to the patients with uterine fibroids, patients with secondary TFI showed significantly higher levels of TNF-α, IL-8, IL-6, and TGF-β1 in the serum samples, whereas the serum cytokine concentrations were statistically significant between patients with primary TFI and the control group (Table 1). Both primary and secondary TFI groups showed significantly elevated levels of TNF-α, IL-8, IL-6, and TGF-β1 in the peritoneal fluid as compared to patients with uterine fibroids (Table 2). However, physiological differences require further discussion.

We next compared the serum levels of TNF-α, IL-8, IL-6, and TGF-β1 between the primary and secondary infertility groups and found no significant differences (Table 5). The cytokine levels in peritoneal fluid samples were statistically significant in statistics but maybe not in physiologically between the primary and secondary TFI groups (Table 6).

Funding section must read:

**Funding**

Yunnan Province Famous Doctor Plan (YNWR-MY-2018-014);

Martin Expert Workstation (2018IC106).

National Natural Science Foundation of China, 31700798;

High talent Project of Yunnan Province, YNQR-QNRC-2018-126.

Reserve talent of Medicine Yunnan Province, H-2017024.

Kunming Medical Union Project, 2018FE001 (−155).

Yunnan Clinical Medical Center for Reproductive Genetic Diseases: ZX2019-01-01.

Health science and technology plan projects of Yunnan Province (NO. 2017 ns214, 2017 ns215, 2017 ns216, 2018 ns0235, 2018 ns0236).

The original article [1] has been updated.
